# Contextualizing Positionality, Intersectionality, and Intelligence in the Anthropocene

**DOI:** 10.3390/jintelligence12040045

**Published:** 2024-04-17

**Authors:** Lisa A. Suzuki, Taymy J. Caso, Aysegul Yucel, Ahad Asad, Haruka Kokaze

**Affiliations:** 1 Department of Applied Psychology, New York University, New York, NY 10003, USA; hk2851@nyu.edu; 2 Educational Psychology, University of Alberta, Edmonton, AB T6G 1H9, Canada; caso@ualberta.ca (T.J.C.); aasad@ualberta.ca (A.A.); 3 Department of Counseling and Clinical Psychology, John Jay College of Criminal Justice, New York, NY 10019, USA; aysegul.yucel@jjay.cuny.edu

**Keywords:** Anthropocene, intersectionality, adaptive intelligence

## Abstract

The geological epoch of the Anthropocene has challenged traditional definitions of what intellectual abilities are necessary to creatively problem-solve, understand, and address contemporary societal and environmental crises. If we hope to make meaningful changes to how our society addresses these complex issues and pave the way for a better future for generations to come, we must advance traditional theories and measures of higher-order abilities to reflect equity and inclusion. To this end, we must address global issues by integrating the complexities of intersectional identities as they impact our understanding of what constitutes intelligence in individuals, groups, and diverse communities. This re-envisioning of intelligence presents new complexities for understanding and challenges for our field beyond the boundaries of what has been previously touted by many disciplines, including psychology. It is an opportunity to re-envision what it means to be intelligent in a diverse global context while also honoring and recognizing the value of difference, positionality, and other ways of knowing.

## 1. Introduction

Several scholars have argued for the urgent need to address racism and other forms of prejudice and discrimination from psychological science, assessment, clinical practice, and research (e.g., Buchanan et al. 2021). Evidence of the disproportionate impact of global climate change, immigration, xenophobia, civil and ethnic conflict, pollution, policing, and the global pandemic on members of marginalized and oppressed communities point to the need for identification and recognition of additional innovative problem-solving strategies, adaptive skills and abilities, and collaborative efforts to reach resolution in context. Defining and operationalizing constructs based on traditional theories or standardized measures is no longer sufficient in an inclusive global context. Instead, we must comprehend various forms of intelligence and their linkage to cultural adaptability and, ultimately, survival by using an intersectional lens to examine how identity impacts access to resources and leads to disparities across realms of experience. This manuscript focuses on what we know about multidimensional identities (e.g., race, ethnicity, socioeconomic status, gender diversity, sexual orientation, immigration, acculturation, generational differences, neurodiversity, and mental health factors) in relation to higher-order cognitive skills and perceptions of what constitutes intelligence based upon current theories and empirical evidence. Our analysis examines the historical foundations of early and contemporary conceptualizations of intelligence from a mainstream psychometric approach while also centering on the importance of diversity, equity, inclusion, and decolonization. We synthesize and provide highlights of the literature related to intersectional domains to illustrate the challenges and complexities of a more nuanced understanding of what intelligence can mean in diverse cultural contexts. 

The search to understand and define the nature of intelligence has led scholars, researchers, and educators in numerous directions. Multiple forms and theories of intelligence have emerged in the literature over the years, and as Boring (1923) noted, intelligence in many settings is defined by the instruments that have been created to measure it. Intelligence tests remain among the most frequently used tools in psychological assessment and have been exported globally as one of the most important contributions of Western psychological practice, at least in part due to how structured and standardized these processes have become. Controversies abound regarding the use of these measures due to a large degree on the racial-ethnic hierarchy of intelligence found repeatedly and consistently in intelligence research based upon psychometric (test-based) approaches that have dominated the field. Clear examples of biased items can be found in tests like the Wechsler Adult Intelligence Scale (WAIS) that rely on individuals having knowledge of U.S. history or sociocultural norms (i.e., items that ask about historical figures like Dr. Martin Luther King Jr.). As our communities become more diverse, our field needs to change to no longer prioritize Western notions of intelligence and instead question how our biases have and continue to replicate hierarchical notions of intelligence that disproportionately impact communities of color. Needless to say, challenges to traditional definitions of intellectual ability during this time of the Anthropocene require a novel understanding of what constitutes intelligence and how it can best be measured and understood.

Intelligence in the Anthropocene brings us to a different level of discourse in terms of global problems and the human potential to address and solve situations that we ourselves have created. The Anthropocene refers to Earth’s geologic age, “in which humans have profoundly reshaped the planet and its biodiversity” (Green 2021, p. 4), leading to global climate change, xenophobia, civil, racial, and ethnic conflict, pollution, increased policing of citizens, a global pandemic, marginalization, and oppression of community groups. More can certainly be added to this list. Not all of these contemporary problems were triggered by the Anthropocene, as the challenges facing our global society are complex given environmental, cultural, historical, societal, political, and economic contexts. Preiss (2022) synthesizes salient highlights of human activity (psychological and demographic) during the past century that have impacted the ecological effects of the Anthropocene and the need to understand and focus on the dynamic and context-dependent human ability as a framework of human intelligence. As part of this need for a dynamic and contextual perspective, we support a movement away from focusing on group differences and instead endorse a broader discourse that is complicated by an acknowledgment and awareness of the complexities of intersectionality (American Psychological Association 2017; Crenshaw 1989; Hays 2016)—age and generation, developmental disability, disability (acquired), religion, ethnicity and race, socioeconomic status, sexual orientation, indigenous group membership, nationality/national origin and language, and gender. For example, while climate change impacts the health and well-being of all people, members of communities with marginalized and oppressed identities—e.g., persons of color, persons of lower socioeconomic status, women, older adults, persons with disabilities—are at greater risk (American Psychological Association Task Force on Climate Change 2022) given the impact of mainstream social, economic, and political power systems. 

Similarly, in *Weathering: The Extraordinary Stress of Ordinary Life in an Unjust Society*, public health scholar Arline Geronimus (2023) coined the term “weathering”, defined as “A process that encompasses the physiological effects of living in marginalized communities that bear the brunt of racial, ethnic, religious, and class discrimination, [and] is critical to understanding and eliminating population health inequity” (pp. 10–11). Geronimus builds on decades of empirical research in public health to explain how weathering systematically and disproportionately impacts people of color. With regard to people of color, she explains, “The repeated or chronic activation of stress processes over years and decades—the measurable physiological stress you feel in the body—has both immediate and long-lasting consequences for physical health and longevity” (p. 37). These claims are corroborated by epidemiological data concerning shortened lifespan, increased rates of infant mortality, and increased incidence and prevalence rates of cardiovascular disease among BIPOC communities, and are now being studied more regularly to address health disparities (Geronimus 2023). 

Sternberg (2021) challenges the reification of intelligence based on our old ways of thinking about intelligence in his historical look at theories designed to explain intelligence and related cognitive abilities. In addition, his critique extends to how intelligence has been operationalized by the tests designed to measure the construct, challenging our field to focus instead on adaptive intelligence in light of the Anthropocene and the need to adapt in response to changing environmental and societal challenges. In view of these changes, scores on IQ tests, as well as the traditional focus on other forms of intelligence (e.g., emotional, social), are less salient. Intelligence must be reconceptualized in light of problems in the real world and contextualized to take into account different ways of knowing that have not been adequately considered historically. To this end, we must factor in our knowledge of historical, social, economic and political realities that have disproportionately impacted some groups more than others. Needless to say, the problems tied to the Anthropocene are coming front and center for “all fields and sectors of society”, and our field has an obligation to address how structural and systemic inequities have impacted our understanding of intelligence (American Psychological Association Task Force on Climate Change 2022). 

Sternberg’s emphasis on creative and practical aspects of intelligence (i.e., successful intelligence) became even more relevant during the COVID-19 pandemic. Throughout the pandemic, low-SES communities had many more hardships to overcome to survive and keep putting food on the table. With the pandemic-related lockdowns, many individuals with financial difficulties had to stay in overcrowded households without access to space, technological devices, and a stable internet connection to work and continue their education. In addition, many low-income college students had to leave their dorm rooms and find new living arrangements while also experiencing food insecurity (Levin 2020). Furthermore, many essential workers at varying levels of professional development (i.e., trainees, early career professionals, etc.) experienced the psychological distress of possibly jeopardizing their health to make ends meet on top of finding childcare and providing their children with daily meals since schools were closed. 

Scholars have documented the disparities that exist given that oppressed communities (e.g., BIPOC; e.g., Carrero Pinedo et al. 2022; Geronimus 2023; Goraya et al. 2023; Suzuki et al. 2022) are differentially impacted at higher rates by historical, environmental, societal, and economic factors. Their work has enhanced our understanding of ourselves, others, and the communities we navigate. The call for our profession of psychology has never been clearer. “Understanding the causes of discrimination in all of its repugnant forms is an urgent goal for psychological science” (Hambrick 2022, p. 9). Understanding systemic racism requires an examination of how “Black, Indigenous, Latinx, Asian, and other non-Black People of color have learned through acculturation, racial trauma, and micro-aggressions to thrive in white spaces and contend with racism” (Liu et al. 2023, p. 244). Systemic racism enables the growth of white dominance and privilege by upholding colonial white supremacy and racial capitalism, resulting in “continued inequities levied against Black and non-Black people of Color” (p. 246). Liu et al. go on to highlight the impact of racial capitalism and the need for the decolonization of research “methodologically and epistemologically” (p. 251), grounding our work in racial-spatial onto-epistemology. Pieterse et al. (2023) highlight the importance of disrupting anti-Blackness and systemic racism in order to focus on culturally responsive conceptual frameworks to increase “relevance and real-world impact” in today’s society and the future. The findings support the movement toward socially responsive research training, attention to systems, ecologies, and social-cultural contexts, and radical healing to address the psychological impact of racism and intersectional experiences of privilege and oppression, with an ultimate impact on training, prevention, and outreach to communities. The authors also note the importance of creating new measures and utilizing innovative and inclusive methods to explore the impact of historical and current systemic and structural racism that informs our understanding of what it means to be intelligent and which groups of people have been historically (and/or are currently) bestowed with this label.

A special edition of the Scientific American (2022) entitled “Science for Social Justice” highlights inequalities and injustice in relation to the political, societal, health care and mental health costs of racism, poverty, oppression, and marginalization. Phillips (2022) work on diversity in work settings notes that “Being around people who are different from us makes us more creative, more diligent, and harder-working” (p. 75). In addition, diversity enhances an understanding of ourselves, others, and the communities we navigate. Hence, skills and abilities associated with “intelligence” are enhanced. The features of diversity that Phillips is referring to include race, gender, disciplinary focus, and any other dimensions that can potentially bring innovative and unique perspectives relevant to the situation and context. A closer examination of the studies cited by Phillips attests to the positive impact of racial diversity on work performance, demonstrations of integrative complexity, and group decision-making. Richard et al. (2003) found in their study that racial diversity enhanced performance, especially for those banks (N = 177) pursuing innovative strategies, giving them a competitive advantage. Moreover, a study by Antonio et al. (2004) addressed small groups of college students consisting of Black and White racial- and opinion-minority members to test the effects of the perceived novelty of contributions to the discussion on participants’ integrative complexity. Findings indicated that varying racial composition and participants reporting racially diverse social relationships were associated with the perceived novelty of group members’ contributions to discussion and integrative complexity (i.e., the degree to which a person’s cognitive style reflects differentiation and integration of multiple perspectives). In a study of group decision-making utilizing a mock trial of a Black defendant, jurors in a racially heterogeneous group examined a wider range of information than all-White groups (Sommers 2006). In addition, White participants considered more case facts, made fewer errors, were more open to discussions of racism, and were more lenient towards the Black defendant when in diverse versus all-White groups. These highlighted studies illustrate the potential benefits of racial diversity in potentially critical aspects of business, perception of integrative complexity, and group decision-making that can be linked to intelligence and intelligent behavior. 

## 2. Diverse Perspectives on the Meaning of Intelligence

It may be argued that early forays into the understanding of intelligence focused on statistical and psychometric definitions, indicating that intelligence is measured based upon the finding of *g* (general intelligence) and sub-abilities. These underlying paradigms centered on positivism and scientific “objectivity” rather than constructivism or social constructionism, which placed power in the hands of the research from the early stages of study conceptualization and design through implementation and dissemination of findings. The positivist approach, by definition, excluded how identities and other socially constructed concepts impact perception and create an imbalanced, hierarchical, and exclusionary lens through which perceived intelligence is examined. This perspective has been criticized, given its reliance on a statistical artifact based upon factor analysis. Tests of intelligence are validated based upon comparison to well-established measures of cognitive ability. The tests are highly correlated with each other and similar in item structure and format. In addition, many predictive ability studies note correlations among IQ, level of education, income, and socioeconomic status (Suzuki and Aronson 2005, p. 321). As White (2000) notes, these “… these are anything but independent variables; they are criteria for one another” (p. 40).

## 3. Definitions of Intelligence

While a number of definitions of intelligence have been presented in the literature, “The Report of a Task Force Established by the American Psychological Association” (Neisser et al. 1996) identifies the concepts of intelligence broadly as follows:

“Individuals differ from one another in their ability to understand complex ideas, to adapt effectively to the environment, to learn from experience, to engage in various forms of reasoning, to overcome obstacles by taking thought”.

(p. 77)

The Task Force notes the limitations of concepts of intelligence given that while “considerable clarity has been achieved in some areas, no such conceptualization has yet answered all the important questions and none commands universal assent” (p. 77). Important areas of consideration in understanding intelligence(s) are highlighted, including psychometric approaches (i.e., intelligence testing), multiple forms of intelligence, cultural variation, developmental progressions, and biological approaches. The Task Force publication represents a step towards elucidating a definition of intelligence; however, as the authors note, there are questions and caveats that remain. These are magnified in the Anthropocene, given the challenges facing our society today, including those with respect to diversity, inequity, and a growing understanding of the challenges of intersectional identities. 

What is indicated is that intelligence is a multifaceted and intricate concept that includes a wide range of cognitive capabilities, problem-solving techniques, adaptability, and the ability to learn and effectively apply information across various contexts. It goes beyond conventional metrics, such as standardized IQ testing, by incorporating a variety of human experiences, including emotional intelligence, creativity, practical wisdom, cultural insights, etc. At its foundation, intelligence entails critical thinking, skillful information processing, and lifelong learning from experiences that allow people to adapt skillfully and resolutely to new situations and difficulties. It behooves us to think beyond fixed definitions, as the construct of intelligence could be seen as a flexible and dynamic set of abilities that enable people to successfully negotiate the complexity of the outside world and make important contributions to society. By acknowledging and valuing the distinctive abilities and talents that individuals and diverse communities bring to improve the common human experience, embracing this broad perspective on intelligence promotes inclusivity and drives the promotion of a more fair and compassionate society.

Further complexities and challenges are evident as we move towards defining intelligence within an ever-evolving global context. Traditionally, intelligence has been defined with an emphasis on cognition or cognitive-based abilities, such as one’s ability to reason, problem-solve, and store a vast amount of knowledge (Suzuki et al. 2019). These abilities have been at the forefront of measuring intelligence, as these skills provide avenues for growth and development (Snyderman and Rothman 1988; Sternberg 2019a). Yet, these aspects of intelligence often fail to consider several nuances that drastically affect one’s ability to adapt in and engage with other contextual factors, such as home environments or cultures, that come with their unique set of rules and demands (Sternberg 2019a; Suzuki et al. 2019; Weiss and Saklofske 2020). 

Research on intelligence continuously concludes that although IQ tests are reliable and valid forms of examining intelligence, these measures primarily test analytical thinking and cognitive reasoning, which only measure a fraction of what intelligence within the current Anthropocene truly is (Kaufman et al. 2022). Psychometric cognitive testing has been critiqued to be a culture, or social situation, of its own that is moderated by “implicit cultural values” that govern how an individual *should* respond to any given item (Ardila 2005, p. 195). These standardized assessment tools continue to be based on ideals that are historically coined by Western communities and scholars, where values of individualism, competition, monolingualism, and self-actualization dominate (Ardila 2005; Ng et al. 2022; Suzuki et al. 2022). Simply put, traditional measures of intelligence focus on ideals that support mainstream ways of learning, understanding, and navigating the Anthropocene (Holden and Tanenbaum 2023). As such, people of color, especially those carrying multiple minority identities, perform consistently poorer on IQ tests and are erroneously deemed to be less intelligent than their counterparts (Berlak 2005; Crenshaw 1991; Hatt 2007; Holden and Tanenbaum 2023). This constitutes an inaccurate attribution for members of marginalized and oppressed communities. 

Within the current Anthropocene, where human efforts and globalization have transformed the world into a melting pot, traditional forms of understanding and testing intelligence may be impotent as it requires substantially more than just Western ideals to conclude who is and is not smart. Complexities are added while defining intelligence when deciphering different forms of intelligence, such as the differences between being “book smart” or “street smart”, where an individual’s unique external environment favors one type of intelligence over the other (Hatt 2007). Contemporary researchers emphasize the practical and creative components of intelligence, also known as successful intelligence, that highlight the role of contextual factors, such as culture, upbringing, race, class, and socioeconomic status, as they inform one’s decision-making skills, logic patterns, and contextualize one’s decisions (Kaufman et al. 2022; Sternberg 2019b; Suzuki et al. 2022). This definition of intelligence goes beyond mainstream values, encompassing multiple forms of intelligence that allow individuals to navigate novel environments through equally complex skills that may not be utilized by dominant group members (Umaña-Taylor et al. 2014). For example, the process of acculturating to a foreign society may require an individual to engage in intricate mental processes, such as code-switching, while maintaining a sense of cultural belonging and identity, values that are inherently fundamental to minoritized groups from collectivist cultures (Umaña-Taylor et al. 2014). As such, the ability to be reflexive, which allows individuals to adapt to the diversifying Anthropocene, could be considered successful intelligence that involves executing creative decision-making skills that lead to practical adaptations to one’s environment (Folke et al. 2021). With this in mind, conceptualizing intelligence as a universal concept that is measured using rigid psychometric tools without taking into account other social-cultural factors questions the validity and fairness of assessment tools as they not only fail to measure complex mental processes related to the contextual factors but also penalize individuals who do not share adhere to Western ideals and expectations (Borgonovi and Ferrara 2020; Holden and Tanenbaum 2023; Ladson-Billings 2006).

Howard Gardner’s (1983) Multiple Intelligence Theory (MI) was one of the earliest theories to move away from the traditional definition of intelligence based on a single general factor (g factor) by suggesting that there is more than one type of intelligence. Gardner also stated that certain types of intelligence, such as musical, interpersonal, and naturalistic intelligence, are not based on cognitive and intellectual traits and are not captured in traditional cognitive ability tests. Sternberg (1984, 1985) proposed the triarchic theory of intelligence highlighting three sub-theories (i.e., contextual—intelligence related to the external world of the individual involving adaptation to the environment, componential—intelligence related to the individuals’ internal world involving learning, planning, execution, and evaluation of intelligent behavior, and the two-facet sub-theory focusing on intelligence as attributed to the external and internal worlds. Later on, Sternberg (1996) proposed the theory of successful intelligence and defined it as one’s ability to lead a meaningful life that is in line with their values by adapting to their sociocultural context, capitalizing on their strengths, and compensating for their weaknesses. Like Gardner, Sternberg also underscored that intelligence could not simply be understood as a cognitive trait as it could include practical and wisdom-based skills—sometimes even more so than cognitive skills—depending on one’s sociocultural context, social locations, and positionality. 

A current focus on numerous forms of intelligence is evidenced in the literature, including emotional intelligence (the ability to understand and regulate one’s emotions and use them as guidance for actions;  Salovey and Mayer 1990), successful intelligence (the ability to adapt to the requirements of the environment; Sternberg 1996), social intelligence (the ability to understand other people and behave wisely in interpersonal relations; Thorndike and Stein 1937), and cultural intelligence (the capability to adapt to culturally diverse settings) (Earley and Mosakowski 2004). The growing literature with respect to each of these forms of intelligence highlights the increasing importance of abilities beyond those captured by mainstream IQ measures and the need to modernize intelligence testing to better support our communities.

## 4. Intersectional Identities and Higher Order Cognitive Abilities

Current professional psychological guidelines and standards emphasize the critical role that intersectionality plays in our understanding of individuals and groups (American Psychological Association 2017; Crenshaw 1989; Hays 2016). In the discussion that follows, we focus on highlighting findings on various identities related to intelligence, including race and ethnicity, socioeconomic status, sexual and gender diversity, immigration, acculturation, generational status, neurodiversity, and mental health factors. 

## 5. Race and Ethnicity

The search to understand the causes and meaning of racial and ethnic group differences in measured intelligence has been a major challenge for scholars for over a century. The focus on group differences, however, leaves out an acknowledgment of consistent findings that within any racial or ethnic group, there is more within-group variability in intelligence than what exists between racial and ethnic groups. An understanding of this phenomenon is complicated, given that what accounts for between-group differences may not be the same as what accounts for within-group differences (Fagan and Holland 2002). The focus on mean differences on measures of intelligence has led to controversial debates, as illustrated in the ruling banning the use of intelligence tests with Black children for the purposes of placement in special education in California (Larry P. vs. Riles). The pursuit to understand race and ethnic group differences in measured intelligence has proven to be one of the most contentious and controversial subjects in the history of psychology and the social sciences. This is due in part to the reification of the construct by scholars and ongoing debates as to the causes, results, and meaning of overall group differences and, in many ways, the profession’s ongoing commitment to use methods of testing that privilege and uphold Western ways of knowing.

Controversies in the race-ethnicity debate have centered around hereditarian vs. environmental and cultural explanations for intelligence differences. Neisser et al. (1996) noted the following:

Several culturally-based explanations of the Black/White IQ differential have been proposed; some are plausible, but so far none has been conclusively supported … Explanations based on factors of caste and culture may be appropriate, but so far have little direct empirical support. There is certainly no such support for a genetic interpretation. At present, no one knows what causes this differential. (p. 97)

Nisbett et al. (2012) indicate that since the publication of the Neisser et al. article, advances in technology, such as imaging techniques, have illuminated the biology of intelligence [i.e., “association between the prefrontal cortex (PFC) and performance on fluid reasoning and executive function and working memory tasks” (p. 141)]; effects of the environment (e.g., schooling, cognitive enhancing pharmaceuticals, physical and cognitive exercise); and the impact of the interaction of genes and the environment [“the heritability of a trait depends on the relative variances of the predictors, in this case genotype and environment” (p. 132)]. The review highlights the complex contributions of genes and environment given that aspects of identity (e.g., age, sex, race, ethnicity, socioeconomic status) impact their potential contribution. For example, the heritability of intelligence test scores differs as a “function of age” and is “not constant across different races or socioeconomic classes” (p. 132). 

As our understanding of social determinants of health, health equity, and systemic and structural violence and oppression have evolved, so too have academic debates around the causes of disparities across all realms of physical and mental health. Ongoing debates centering around hereditarian vs. environmental or cultural explanations continue despite evidence of the interaction between genes and environment noted earlier. A recent meta-analysis focused on racial and ethnic group differences in the heritability of intelligence (Pesta et al. 2020), including an overall sample of participants of 84,897 Whites, 37,160 Blacks, and 17,676 Hispanics residing in the U.S. The authors focused on examining if the heritability of intelligence varies by racial or ethnic group, specifically if those groups living in disadvantaged environments (e.g., lower SES) reflect lower “heritability and higher environmentality” (p. 101). Overall findings indicate that heritabilities did not substantially differ by race or ethnicity, indicating a lack of support with predictions that potential for adaptive functioning will more likely be expressed in supportive and nourishing environments (i.e., the Scarr–Rowe hypothesis cited in Pesta et al. 2020). Challenges to these findings included that the authors were racially motivated in their questioning and the study was “poorly executed”, leading to questioning the peer review process and “rigorous editorial judgment” of the journal *Intelligence* (Giangrande and Turkheimer 2022). The meta-analysis was criticized on the basis of “severe theoretical, methodological, and rhetorical flaws” (p. 696).

The search to understand the racial and ethnic divide in intelligence has been explored on a global scale, as evidenced by (Lynn 2019; Lynn and Vanhanen 2002), whose work examines intelligence associated with the per capita income of different nations of the world. In summary, he writes the following:

… national IQs were significantly causal to educational attainment, per capita income, economic growth, income inequality, cognitive achievements, political institutions (e.g., democracy, market economy), happiness, religion, health, nutrition, crime and fertility and significantly the result of differences in race and ethnicity morphology and physiology (e.g., brain size), geographical location (latitude) and genetics.

(Lynn 2019, p. 127)

This work has been criticized for hereditarian interpretations of racial and ethnic group differences in intelligence, leading to cancellations of speaking engagements, resignations, firings, attacks, lawsuits, demands for dismissal, etc. The far-reaching impact of these consequences has shaped how scientists and researchers study intelligence, report their findings, and interpret the meaning of race and ethnic group differences in intelligence. In determining IQs for different nations, Lynn includes attention to aspects of intersectionality beyond just race and ethnicity (e.g., indicators of socioeconomic status (e.g., income), religion, national origin, etc.). 

The question of the malleability of measured intelligence is one in which the answer remains in flux. Beliefs around whether intelligence is fixed and innate or more malleable can be attributed to experience and to differential beliefs related to fluid intelligence (i.e., reasoning involved in solving novel problems) and crystallized intelligence (i.e., knowledge and skills; Sun et al. 2021). Sun et al. address people’s reasoning about the malleability of intelligence and its inheritance in relation to the definition of intelligence (crystallized and fluid) under consideration. Their findings indicate that participants viewed “crystallized intelligence is most malleable, followed by general intelligence, then followed by fluid intelligence” (p. 824). Participants’ beliefs also suggested that “adoptive parents (i.e., environment) would have a greater influence on a child’s crystallized intelligence, but that biological parents (i.e., biological inheritance) would have a greater influence on a child’s fluid intelligence” (p. 824). Hence, the authors conclude: “… fluid intelligence was viewed as not just more fixed, but also as more innate and determined at birth. Crystallized intelligence in contrast was viewed not only as more changeable, but also as more influenced by experience” (p. 825). These findings align with past research indicating that fluid abilities are less influenced by experience and “more biological based than crystallized intelligence” (p. 825). A key component of this perspective relevant to our discussion is that race and ethnic group differences have been noted more in terms of fluid abilities in comparison to crystallized. Our understanding of race and ethnic group differences in intelligence is highly dependent upon the explanatory perspective the scholar brings to the findings. 

The complexities of understanding the measurement of intelligence and what appeared to be gains in intelligence over time were challenged by findings of the Flynn Effect, given the application of obsolete norms.

“The inflation of IQs because of obsolete norms led to inflated estimates of the effects of interventions, adoption, and aging, and also misdiagnosis of whether individuals had met IQ cutting lines that affected everything from the administration of the death penalty to who should benefit from special education”.

(Flynn 2020, pp. 940–41)

Flynn’s work attests to the “life history of IQ gains”, noting that while hypotheses would lead us to believe that the industrial revolution, modernity, smaller families, greater understanding of cognitive development of young children, better schooling, etc., lead to higher IQ scores, in reality, “very advanced nations that have been highly industrialized for more than a century may be showing signs of IQ decline” (p. 950). While the 20th century brought attention to alleviating poverty, improving formal schooling, increasing cognitive demands in the job market, liberating women, etc., we face greater challenges in the 21st century. Flynn concludes that what is needed is “what Aristotle called ‘practical wisdom’, a collective effort to humanize our societies with critical intelligence and knowledge at a premium” (p. 959). 

## 6. Socioeconomic Status

As alluded to earlier, the relationship between growing up in low socioeconomic status (SES) households and obtaining lower scores on standardized intelligence tests has been well-established (Duncan et al. 2017; von Stumm and Plomin 2015; Carman and Taylor 2010). Studies documented that the environmental and psychosocial factors that lead to differences in IQ scores of low-SES and middle- or high-SES children include low parental education (von Stumm and Plomin 2015), minimal exposure to a child-directed speech (Rowe 2008) and cognitive stimulation in the home environment (Rindermann et al. 2010), lack of access to enriched educational opportunities (Lucas 2001), and experiencing psychological distress as a result of the financial strain in the household (Ursache and Noble 2016). While this disparity urged researchers and policymakers to focus on the development of interventions (e.g., the Head Start program) to boost the test scores of children from low-SES communities and help them “catch up” with their middle-class peers (Anderson et al. 2003), a group of scholars pointed out that the primary issue lies in the way intelligence is traditionally defined and the psychometric tests based on the traditional definition of intelligence fall short when evaluating individuals outside the white, middle-class, and Western groups (Helms 1992; Ford 2004; Reynolds and Suzuki 2012). 

Sternberg (2019a) emphasized the importance of adaptation skills when understanding intelligence by stating, “Place many of us even in an inner-city ghetto overnight, and we might not survive until the next morning to tell our experiences, whereas ghetto children … much younger than we are, likely would be there the next morning to tell the tale” (p. 3). Using other words, youth (in this case, those living in impoverished and resource-scarce areas) will fare better than their privileged (often White) counterparts in similar contexts. According to Sternberg, even though the cognitive processes underlying intelligent behaviors are consistent across all groups, the manifestation of intelligent behavior vastly varies depending on their sociocultural and economic context. For instance, receiving high scores in math might be a highly adaptive—and therefore intelligent—behavior for a child from a middle-class, Western household, and it could potentially provide the child with the appraisal of their parents and teachers on top of different educational opportunities. On the other hand, performing well in educational settings might not be as necessary or perhaps has been historically infrequent or not possible and, therefore, may not hold the same value in families experiencing chronic financial stress. Instead, children who can incorporate other skills that are not tapped into in the conventional definition of intelligence, such as taking care of younger siblings or taking care of additional housework when a parent has to work overtime and navigate keeping themselves safe when walking home from school each evening in a neighborhood with high crime rates, could be seen as wise. Youth may also serve as cultural brokers for their immigrant parents and elders, given their understanding, adjustment and acculturation to new societal systems and language, often through exposure to the educational system.

Ellis et al. (2022) also emphasized the need to recognize the adaptive skills of individuals who chronically survive harsh living conditions, including poverty, as a part of our understanding of intelligence. They conceptualized the skills needed to survive and thrive in unfavorable and unstable living conditions as a form of adaptive intelligence and named it “hidden talents”—referring to the often unnoticed nature of these skills in Western educational settings. According to Ellis et al. (2022), these skills include being acute at recognizing and remembering negative information, having the mental flexibility to switch between doing the task at hand and paying attention to the environment, having increased sensitivity to interpersonal cues, having the ability to think outside the box, and coming up with solutions to stressful life situations by “extracting” resources. These statements align with the findings of Abrams and Terry (2014), who conducted in-depth interviews with formerly incarcerated men of color to understand the specific skills they developed to survive in difficult circumstances after being released from prison. The results indicated that the men in their sample developed particular skills such as avoiding potentially dangerous crowds and situations, being mindful about the level of risk-taking, and running and hiding when confronted with unsafe circumstances. Therefore, in light of successful intelligence, these skills and associated behaviors displayed by these men are crucial for survival in their current environment and would be considered intelligent behavior more than solving a complex math problem. 

Nunes et al. (1993) conducted a set of studies in Recife, Brazil, which showed that while Brazilian street children were able to do math in their everyday lives as needed, they were not able to translate their skills to paper-and-pencil tests in classroom settings. This finding shows that not only the adaptive skills—in other words, intelligent behavior—that are required to survive and thrive in various cultures are different from each other, but also the environment in which those skills are manifested (e.g., in the street vs. in school) can also vastly differ based on the culture. Thus, while Kenyan and Brazilian children were able to master skills that are highly adaptive and potentially life-saving in their environment, they would perhaps be perceived as unintelligent and treated accordingly (e.g., be placed in special education classrooms due to their low test scores) if they immigrated to a western country. 

Overcoming hardships and surviving requires various skills, such as tolerating systemic threats and uncertainty of one’s status and safety, advocating for your rights, combatting and dismantling structural and systemic discrimination, finding creative solutions to unanticipated problems, and building and maintaining strong interpersonal relationships that one can rely on during difficult times. The manifestation of these skills should be interpreted as highly adaptive, socially astute, and intelligent behaviors from a successful intelligence perspective but are certainly not taken into account by the traditional understanding of intelligence. Carrero Pinedo et al. (2022) note the importance of acknowledging the resilience of Black, Indigenous, and People of Color (BIPOC) in their examination of the experience of trainees and members of minoritized communities. They note that BIPOC “hold multiple intersectional identities that are exacerbated by the injustices they encounter in their professional paths” (p. 140), which has relevance for our evaluation of what constitutes intelligence and ability. When BIPOC are exposed to numerous hostile threats in their professional environment, they are faced with recognizing the harms they are subjected to, having to find socially acceptable ways of responding to people who deny their experiences, and still over-producing and overcompensating in their performance to buffer against likely discrimination they will continue to face. They often engage in these self-preserving techniques as a way of surviving a system of learning that does not recognize or celebrate the richness of their diversity and the adaptiveness of their coping mechanisms. For these reasons, it is critical that professionals work to address historical distributions of power, systems of privilege and oppression and legitimize the experiences of members of BIPOC communities. Furthermore, (Carrero Pinedo et al. 2022) assert that the field of psychology is “uniquely positioned to transform how science and practice informs, builds, and sustains equitable systems for trainees and the public” (p. 140). 

## 7. Sexual and Gender Diversity

Conceptualizations of intelligence are shaped and limited by several structural factors, like other constructs used to capture individual differences and performance: tools available for measurement, individual interpretation of performance, and professional consensus of standardized scoring on assessments. Similarly, individual-level characteristics, including language spoken, performance anxiety during an assessment, cultural values associated with intelligence, and internalized biases about testing (e.g., model minority issues, stereotype threat, dominant narratives about perceptions of intelligence, etc.) can have an impact on how intelligence is understood (Rindermann et al. 2020; Roberts et al. 2020). Historical research has often produced science that characterizes minoritized groups as inferior in outright measures of IQ, academic achievement, and general cognitive tasks (Cirillo et al. 2020; Hartmann et al. 2013; Roberts et al. 2020). Often these disparities reflected in the research literature have focused on race (Roberts et al. 2020) and gender and sexual orientation (Cirillo et al. 2020; Jiang et al. 2020). 

Although contemporary and younger generation psychologists have persisted in challenging historical notions of how we operationalize and privilege certain types of intelligence, there is limited research and empirical evidence that examines how intelligence is understood specifically among sexual and gender minorities that is inclusive of transgender and gender diverse individuals. These gaps in the literature are impacted by the complexities and nuances of sociopolitical identities, limitations in suggested guidelines for how to report gender in research (e.g., prioritizing sex assigned at birth as opposed to sex assigned at birth in addition to affirmed gender identity) (Bowleg et al. 2023; De Castro et al. 2016; Heidari et al. 2016), non-inclusive norms established for standardized measures of intelligence (Cicchetti 1994; Ford 2004), and poor sampling strategies (i.e., limited representation among convenience samples, not implementing recruitment strategies that target minority participants, etc.) (Ford 2004; Roberts et al. 2020), and other methodological challenges.

A recent study conducted by Rindermann et al. (2020) collected data on the identities and views of intelligence experts across a variety of topics, including research on intelligence testing and IQ, the media, and controversial topics in the field. Their findings shed light on the disparities in representation among experts on intelligence, reflecting that the field is dominated by cisgender men from Western backgrounds, and their views reflect biases informed by their political affiliations and ideology (Rindermann et al. 2020). For instance, their data indicated that participants who identified as conservative were more likely to also endorse beliefs about gender and racial differences in intelligence, as well as support the integrity of IQ testing (Rindermann et al. 2020). When considering the impact of situated domains of knowledge on the development of science (Haraway 1988), we must consider how biases inform how constructs are conceptualized. 

While concerns are raised regarding how intelligence may be inaccurately portrayed in minoritized communities, work by many scholars and researchers has contributed to our understanding of intelligence and intersectionality that apply to sexual and gender diversity. Some researchers and scholars have raised meaningful questions about how the lack of representation among leading experts from marginalized communities might also limit diverse perspectives, contributing to stagnation in the field, at times through rejection and censure of views and data that challenge historical norms (Hartmann et al. 2013; Medin 2017; Rindermann et al. 2020; Roberts et al. 2020). Others have begun to question the structural integrity of how intelligence testing is conducted, particularly focusing on how high-stakes environments can disproportionately disadvantage individuals from under-resourced and impoverished backgrounds (Roberts et al. 2020). Other relevant questions that need to be examined are related to how these constructs are defined and measured in ways that do not benefit some groups over others.

A deeper understanding of minority stress and structural and societal oppression faced by individuals who have minoritized gender and sexual orientation tells a striking story (Cyrus 2017; Kelleher 2009). LGBTQ individuals often have to develop adaptive strategies to navigate prejudice and discrimination across social contexts and cope with the distress produced by constant exposure to minority stress (Fulginiti et al. 2021). For some individuals, these strategies can take the form of identity concealment and code-switching (Allen and Lavender-Stott 2020; Breslow et al. 2015; Carrero Pinedo et al. 2022; Gonzalez et al. 2021; Livingston et al. 2016), which often require higher levels of emotional intelligence, resourcefulness, and social astuteness, in addition to other compensatory behaviors. Despite the wide recognition of the effect minority stress has on sexual and gender minorities, in addition to other identity-based sources of marginalization (e.g., racism, colorism, texturism, featurism, xenophobia, etc.), the use of such skills and strategies should be reflected in measured intelligence. Doing so might further debunk pseudoscience concerning the inferiority of some groups, particularly individuals from diverse backgrounds who also hold other minoritized identities, while also encouraging researchers in this area to question biases in their research.

One example illustrating the limitations and implications of current professional practices is evident as no intelligence, personality, or neuropsychological tests have been normed or validated on the transgender population (Keo-Meier and Fitzgerald 2017). Yet, transgender people have undergone psychological testing as a standard part of presurgical evaluation and treatment to access gender-affirming transition-related care for decades. Indeed, “mood and cognition are factors considered in presurgical assessments” (p. 54). In addition, no professional guidelines exist for interpreting test data (e.g., intelligence) in reference to those with a transgender identity. Empirical studies suggest that there are differences between genders (cisgender or transgender, but not taking into account all transgender and gender diverse identities) in relation to cognitive factors. Specifically, strengths are noted for cisgender women in terms of verbal intelligence, while cisgender men have higher spatial intelligence. In their review, Meier and Fitzgerald hypothesize that cross-sex hormone administration may impact cognition. Studies of hormone therapy and cognition have indicated increases in spatial ability associated with testosterone, but the findings are not conclusive over multiple studies. The review suggests overall that testosterone is related to increases in spatial skills, however, effects on verbal ability are unclear. Structural differences in pretransition populations are also an area of study that may impact the understanding of cognitive changes. Hormone therapy has also been found to impact mood, which in turn may negatively influence cognition.

It is important to note that there is growing attention to the use of behavioral measures with transgender and gender-nonconforming children and adolescents, indicating the need for the inclusion of nonbinary youth in clinical samples (Rider et al. 2019). Preliminary data suggests that performance on measures like the CBCL may be influenced by gender dysphoria, minority stressors, and interconnected systems of oppression. Further evidence is noted, given findings from the Youth Survey Report (YSR) documenting higher rates of depression, suicidality, self-harm behaviors, and eating disorders in comparison with peers for transgender youth (Connolly et al. 2016). This information supports the need for culturally sensitive and gender-affirmative testing practices, including a focus on resilience, as well as cognitive strengths and abilities.

## 8. Immigration, Acculturation, and Generational Status

As of 2019, 44.9 million immigrants have been residing in the U.S. (Esterline and Batalova 2022), making up 13.7% of the population. Yet, similar to other minority groups, immigrant experiences are often overlooked and disregarded in the context of intelligence, and immigrants are stripped of opportunities enjoyed by their U.S.-born counterparts in various settings. For instance, English Learner (EL) students—mainly students born outside of the U.S., born in the U.S. but had immigrant parents, or grew up in non-English speaking neighborhoods—are underrepresented in gifted and talented education (Mun et al. 2020) and overrepresented in special education classes in the U.S. K-12 education system (Ford 2004). Undoubtedly, how intelligence—and giftedness, for that matter—is defined and measured based on the norms and values of white, Western, middle-class, and U.S.-born groups significantly impacts these disparities (Ford 2004). The lack of representation of EL students in gifted education is significant not only because it signals the biased nature of the standardized IQ tests used to evaluate students for giftedness but also because the selection often includes a nomination process where teachers identify students with the potential to be tested. Thus, this underrepresentation could inform us about teachers’ perceptions of EL students’ intelligence. For example, Costello (2017) conducted observations, interviews, and focus groups in elementary and middle schools in Florida to explore the teachers’ nomination process regarding EL students. The results indicated that the teachers believed that the EL students’ lower oral language skills and demonstration of traditional cultural practices in school settings often “masked” their talent, causing them to go unnoticed during the gifted identification process.

In another study by Harradine et al. (2013), teachers were asked to observe students for 3 to 6 weeks by using Teacher’s Observation of Potential in Students (TOPS)—a measure developed to identify academically talented students of color—rather than solely based on their judgment. Participant reflections after the study indicated that the teachers believed that without using the TOPS, they could have overlooked identifying the potential of 22% of students of color. Participants also noted that oral language skills were one of the barriers during the identification process—especially for Latinx students. 

Unfortunately, the findings of Costello (2017) and Harradine et al. (2013) regarding how EL students’ limited oral skills and cultural values mask their intellectual potential in educational settings are not surprising. Numerous other studies documented that what is deemed as intelligent behaviors in various non-Western cultures is in no way, shape, or form included in the Western understanding of intelligence. For example, with the intent to show that practical intelligence—defined as one’s ability to acquire knowledge from daily experiences and use it to solve practical problems of their environment as they emerge—is separate from academic intelligence, Sternberg et al. (2001) conducted a study with 85 children between the ages of 12 to 15 in rural Kenya. The results showed that while Kenyan children received low scores on the tests measuring academic intelligence, they exhibited high practical intelligence by demonstrating knowledge about different natural herbal medicines and which ones to use for various illnesses, such as parasitic infections, that are common in their environment. 

Moreover, immigrants often face numerous obstacles in the U.S., such as stigmatization, poverty, lack of quality health care (Derose et al. 2007), loss of social status, occupational downgrading (Euteneuer and Schäfer 2018; Fernando and Patriotta 2020), uncertain life conditions (e.g., fear of deportation if undocumented) (Joseph 2011), lack of social support system and difficulties adapting to the language and culture of their host country while maintaining their own cultural values (Caplan 2007). However, studies suggest that despite these adversities, Latinx individuals who recently arrived in the U.S. typically report similar or better mental well-being than their U.S.-born Latinx peers (Alegría et al. 2008). Even though the exact reason for this phenomenon—called the “immigrant health paradox”—is unknown, it is plausible to infer that the immigrant youth seem to have a specific set of skills or habits (e.g., better health practices such as consuming less alcohol) (Abraido-Lanza et al. 1999) that enable them to not only adapt to challenging life conditions but also thrive in a new country after their lives have been uprooted. 

Acculturation has also been linked to language proficiency (including speaking without a foreign accent) and familiarity with intelligence test formats. Acculturation refers to a “dynamic process of change and adaptation that individuals undergo as a result of contact with members of different cultures” (Rivera 2008, p. 76). The process of acculturation is often characterized by stress given cultural, social, and psychological contexts and the resulting experiences of the immigrant or refugee (Short et al. 2010). Studies of acculturative stress and internalization of mental health problems have been associated with depression, anxiety, and poor academic behaviors in Latinx students’ academic performance (Albeg and Castro-Olivo 2014). This is a critical finding, considering that Latinx students who speak English as a second language often experience considerable pressure to lose their accents and other markers of culture, ethnicity, and race (Rosa 2019).

Research indicates that even when everything else is held constant, immigrants tend to be deemed less intelligent if they speak English with a “nonnative accent”, a clear example of implicit bias and preference for native-English speakers. For example, in a study by Nelson et al. (2016), participants were asked to listen to several recorded passages read by either North American or Spanish-accented English speakers. Afterward, they were asked to rate their perceived competence of the person who read the passages. The results indicated that Spanish-accented recorders were rated less intelligent than North American-accented recorders. The findings of this study highlight that people who speak in nonnative accents—which in most cases are immigrants—are deemed less intelligent by others during daily interactions, which could adversely impact various aspects of their lives, from their employment opportunities (Chakraborty 2017) to how much they feel respected by others (Derwing 2003). 

## 9. Cultural Neuroscience and Neurodiversity

Understanding intersectionality in neuropsychology, cultural neuroscience, and neurodiversity offers exciting new directions for the study of cognition and behavior, as well as health promotion. Glimpses into these relatively new areas offer important insights into our understanding of intelligence in the Anthropocene, given the importance of survival and adaptation—how can we maintain core aspects of intelligence assessment despite their historical limitations while also honoring differences among people and their adaptive potential? Both constructs challenge our mainstream way of thinking about intelligence from interdisciplinary perspectives and require our attention to ensure that we develop innovative ways of being more inclusive to not only address historical disparities resulting from flawed ways of understanding intelligence but to ensure that as a field we can provide comprehensive ways of understanding the ever-evolving nature of intelligence and adaptation. 

Cultural neuroscience merges the fields of anthropology, cultural psychology, neuroscience, neurogenetics, and population genetics (Fujii 2023). In his review, Fujii cites how current theories “support that the environment influences brain organization, functioning and cognition … intersectionality principles would indicate that societies have social hierarchies based upon the characteristics, identities, or behaviors of its people” (p. 156). Social hierarchies privilege particular groups and disadvantage others (i.e., nonprioritized minoritized groups). He identifies institutional biases for brain development and organization in terms of education, economics (e.g., poverty), and health disparities. Examples of support for these findings are yielded in neuroimaging studies indicating a positive relationship between years of education and cortical volume; poverty is related to a reduced cortical surface area, thickness, and overall volume in the frontal and temporal areas related to attention, language acquisition, executive functioning, memory and emotional regulation; and chronic discrimination has been identified as “neurotoxic and associated with cognitive deficits” (p. 159). 

Bhui (2018) provides a summative review of the literature on the development of the field of cultural neuroscience. 

The central premise of cultural neuroscience is that culture and biology co-evolve, both being adaptive, and that just as genetics, biological vulnerabilities and affordance give rise to specific abilities within which culture emerges, so cultures that permit populations to flourish are not only selected for survival value but also shape biological affordances. (p. 57).

“Cultural influences impact the nature of encoded neural responses in the brain, including constructs related to emotion (e.g., innate emotions, socially constructed emotional experiences, cognitive and psychological). The need to move from a focus on deficits and pathology toward an understanding of the … power relationships that may be at play when one discipline or another asserts superior benefits and gains…Cultural neuroscience offers hope and optimism, and opportunity to better understand how culture is encoded in the body and brain, and how cultural biology might transform the way culture is understood and how cultural psychiatry evolves …”.

(pp. 56–57)

A focus on how culture systematically influences the brain has implications for how we understand the nature of intelligence and intelligent behavior (Goraya et al. 2023). Kitayama and Salvador (2017) cite the literature indicating that “through socialization, neural networks are plastically formed and modified through various rewards and reinforcements over time and become patterned after cultural beliefs, values, and practices” (p. 844). By the same token, active engagement and feedback from the environment can lead to change in neural networks. The authors note the importance of the continual study of performance-based measures of culture and neural counterparts that may lead to important breakthroughs in understanding the nature of culture. 

Neurodiversity. Neurodiversity is perhaps the newest form of intersectionality that highlights the “uniqueness of *all brains*” (Botha and Gillespie-Lynch 2022), acknowledging that people process and interact with their environment and people around them in different ways. Neurodiversity refers to “individuals with differences in brain function and behavioral traits as part of normal variation in the human population…Neurodiversity is about uncovering the strengths of neurodiverse individuals and utilizing their talents to increase innovation and productivity of society as a whole” (https://med.stanford.edu/neurodiversity.html, accessed on 20 March 2024). Recognition of neurodiversity has been supported by those diagnosed with Autism, Attention Deficit/Hyperactivity Disorder (ADHD), various forms of learning disabilities (e.g., dyslexia, dyscalculia), and their allies. While neurodiversity impacts all communities, the research literature on health disparities signals a number of ways in which BIPOC people face structural and systemic barriers to care, above and beyond non-racialized groups (Bowleg et al. 2023; Jones et al. 2020; Goraya et al. 2023; Hotez and Hudson 2023). People with neurodivergent identities must be understood through the lens of multifaceted identities and contexts within a framework that acknowledges systemic forms of oppression and marginalization experienced by minoritized communities (Goraya et al. 2023; Hotez and Hudson 2023; Jones et al. 2020). 

Thinking more broadly about neurodiversity, Botha and Gillespie-Lynch (2022) cite literature indicating the following:

“Although the idea of neurodiversity has been adopted to varying degrees by other disability communities … intellectual and learning disabilities … who should be included within the neurodiversity movement remains highly contested.”.

(p. 96)

The authors note that Autism is reified in the DSM-5 as a “social-communication disorder which *must* involve some degree of suffering, and which, according to some, should be prevented, cured and eradicated” (Botha and Gillespie-Lynch 2022, p. 93). Neurodiversity implies that Autism and other conditions associated with neurodiversity are part of a “valuable minority identity that needs no cure” (Kapp et al. 2013; Kapp 2020; cited in Botha and Gillespie-Lynch 2022, p. 94). The literature highlights that neurodivergent individuals must be understood in terms of their multifaceted identities, contexts, and communities. 

Studies indicate that the intelligence of neurodivergent individuals, like those with Autism, have been underestimated by traditional measures (Goraya et al. 2023). In addition, autistic individuals are more likely to experience early mortality, suffer from depression, anxiety, and post-traumatic stress disorder, and have their gender identity “dismissed” (Botha and Gillespie-Lynch 2022, p. 99). The minority stress model highlighting the cumulative effect of social stressors on marginalized and oppressed groups in our society can be applied to members of the neurodiversity movement. 

Studies of high-functioning students with Autism/Asperger Syndrome indicate that their performance on intelligence and other cognitive-based measures yield discrepant abilities across subtests. These individuals generally perform well on tasks that require sustained attention, recognition of facts and information processing and less well on tests requiring shifting of attention, flexible thinking, and complex information processing (Sansosti et al. 2010). The existence of cognitive strengths and limitations means that an overall IQ score may not be indicative of the intellectual potential of the individual with Autism. Therefore, caution is required in the usage of traditional mainstream measures of intelligence. In recognition of the need to provide and broaden opportunities for neurodivergent individuals to thrive and reach their potential, specialized programs have been developed (e.g., Google and the Stanford Neurodiversity Project have (https://www.myautism.org/news-features/new-google-program-to-hire-people-with-asd, accessed on 20 March 2024). The other is the Nest Support Project at NYU, which highlights the vision “of a world that authentically embraces its inherent neurodiversity”, recognizing and honoring the “complexities and intersectionality of each individual’s social identity” (https://steinhardt.nyu.edu/metrocenter/nest/values-nest-support-project, accessed on 20 March 2024). 

In addition to Autism, one of the most frequently identified areas of neurodiversity is ADHD. A meta-analysis by Frazier et al. (2004) suggested that individuals with Attention Deficit/Hyperactivity Disorder (ADHD) tend to demonstrate overall lower intellectual abilities than those without ADHD. Similarly, people with ADHD often experience impairment in executive functioning, such as problem-solving issues skills and poor working memory (Martinussen et al. 2005). 

A study by Climie et al. (2019) showed that even though children with ADHD scored lower on specific subscales of the emotional intelligence (EI) test, their overall EI scores were not statistically different from their peers without ADHD. Moreover, Schirduan and Case (2004) conducted a study with 87 students from grades 2 to 7 who attended a school where the curricula were designed in the light of the multiple intelligences theory of Gardner (1983) rather than the traditional public schools where the curricula were primarily based on linguistic and logical-mathematical intelligence. The findings of the study demonstrated that even though students with ADHD often perform worse than their peers without ADHD in traditional school settings, they showed average performance if the curricula are designed in a way that takes the strengths of students with different cognitive profiles into consideration by incorporating the multiple intelligence theory. The findings of Schirduan and Case (2004) also showed that more than 50% of students with ADHD in the study stated that their strongest forms of intelligence were naturalist and spatial intelligence, which largely go unnoticed in educational settings. Once again, both studies highlight that IQ tests solely based on intellectual traits fall short when different groups, such as people with ADHD, are assessed. Though this section on neurodiversity focuses primarily on Autism and ADHD, findings indicate the need for a more inclusive, adaptive, and intersectional understanding of cognitive abilities and examination of how we assess intelligence in these diverse communities. 

## 10. Mental Health Factors

Numerous studies documented that individuals with psychiatric disorders tend to obtain lower scores on conventional IQ tests. For example, Viezel et al. (2015) compared the IQ scores of 120 children in foster care who experienced trauma in the form of maltreatment in the past to those without a maltreatment history. The findings indicated that children with a history of trauma had lower overall cognitive ability scores than the comparison group. A review by Castaneda et al. (2008) also suggested that the presence of the symptoms of depressive and anxiety disorders might be linked to cognitive impairments in young adults. Most mental health diagnoses include some cognitive impairment that could interfere with the individuals’ ability to perform well on standardized IQ tests. For example, two symptoms of depression are memory and concentration problems (American Psychiatric Association 2013) and high levels of physiological arousal and intellectual and behavioral reactions caused by anxiety, especially performance anxiety, lead to decreased performance on academic and neuropsychological tests (Hopko et al. 2005). 

Furthermore, from the successful intelligence perspective, people who experience symptoms of a psychiatric disorder often exhibit unique skills and adaptive behaviors to navigate the challenges brought on by their conditions. Besides the difficulties related to the symptoms of their illness, they often experience stigma and discrimination in various aspects of their lives, from education to employment to interpersonal relationships. In many cases, they have to tolerate uncertainties regarding the course of their illness. However, many people with psychiatric disorders show resilience, which requires adaptive skills, including but not limited to accepting the reality of their condition and finding ways to move forward with this new normal. For instance, the findings of a study by Tuffour et al. (2019), where 12 Black African individuals who used mental health services in England and are currently recovering from mental illness were interviewed, showed that while participants stated that they were initially devastated by their diagnosis, they could eventually practice acceptance and continue their lives with their illness. 

In another study by Edward et al. (2009), participants who had experienced mental illness were interviewed to explore how resilience played out in their lives. The general themes that emerged in the interviews were summarized as “viewing life from the ridge with eyes wide open” by the authors (Edward et al. 2009, p. 592), emphasizing the fact that the participants talked about being able to continue with their lives by incorporating valuable skills and behaviors such as helping others, practicing acceptance, maintaining hope, creating some balance in their lives, and keeping up with their daily tasks despite uncertainties and risks regarding their futures. These skills and behaviors demonstrated by people with mental health difficulties could be described as highly intelligent from a successful intelligence perspective while not acknowledged in the conventional understanding of intelligence. 

## 11. Summary and Conclusions

Contemporary issues facing our society today demonstrate the limitations of traditional definitions of intelligence solely based on cognitive and intellectual traits and the need for revision and reconceptualization of what it means to be intelligent in the Anthropocene. The increasing complexity of our historical, societal, educational, and environmental landscape presents innumerable challenges that can no longer be addressed solely by groups deemed “smart” due to their high scores on standardized IQ tests or academic excellence. Instead, we need, more than ever, individuals with diverse perspectives to bring about innovative, creative, and sustainable solutions. 

As mentioned throughout the manuscript, many scholars have been working on revising the narrow and outdated definition of intelligence in the past few decades by proposing alternative forms of intelligence in relation to mainstream theories (e.g., multiple intelligences, social intelligence, emotional intelligence, successful intelligence, adaptive intelligence, cultural intelligence). Some researchers have also been drawing attention to the fact that standardized psychometric tests are based on the norms and values of white, Western, cisgender, straight, middle-class, U.S.-born, developmentally, psychologically, and physically “healthy” groups and thus are biased toward marginalized communities. In addition, this manuscript has briefly highlighted the scholarly debate and controversies surrounding the meaning and interpretation of race and ethnic group differences in intelligence. Recent publications (e.g., meta-analyses Pesta et al. 2020; Giangrande and Turkheimer 2022) serve as contemporary examples of how empirical evidence on intelligence can be interpreted from multiple and conflictual perspectives.

Ceci’s (1996) support of the bioecological framework on intelligence highlights the complex relationship between culture, abilities, intelligence, and context with clear implications for intersectionality. Ceci highlights the importance of proximal processes, i.e., “sustained interactions between a developing organism and the persons, symbols, and activities in its immediate environment. To be effective, these processes must become progressively more complex and interactive over time.” (p. 245).

There have also been debates regarding how making educational and occupational decisions based on the results of standardized cognitive ability tests should be revised, changed and, in some cases, eliminated as these procedures continue to perpetuate existing inequalities in our society. As noted throughout this manuscript, the Anthropocene calls for the importance of understanding intelligence from an intersectional and health equity-informed lens that recognizes the relationship between identities and social locations individuals hold and historical power hierarchies that impact our understanding of this complex construct in context. Reviews of the literature on intersectionality with respect to race and ethnicity, socioeconomic status, sexual and gender diversity, immigration, acculturation and generational status, neurodiversity, and mental health factors highlight the complexities and challenges of understanding intelligence with respect to diverse cultural, historical, societal, economic, and political contexts. We also note that intersectional identities and contexts are not static but dynamic requiring flexibility in adaptation over time. While the alternative theories of intelligence and debates regarding the use of psychometric tests are undoubtedly a step in the right direction, the researchers in the field of intelligence must adapt to how our society’s ever-changing nature and needs might continuously transform our understanding of intelligence and be committed to keeping to pay attention to how intelligence is manifested in different sociocultural contexts and various marginalized communities as represented in a focus on intersectional identities. We have addressed only some of the features of intersectionality; the landscape of survival and adaptation in the Anthropocene is uneven and characterized by glaring disparities. Only with awareness and commitment will new windows of opportunity emerge to bring people together to bring about the much-needed change in our society that is continually plagued with issues including systemic racism, xenophobia, anti-LGBTQ rhetoric, a global pandemic, prevalent mental health crisis, and global climate change. 

We end with a note of caution by Ceci:

“The journey to understanding individual and group differences in intellectual functioning has been a long, winding path, trod by many scholars traveling from different and distant scientific climes. Until the dust settles and a common destination becomes visible, scientists should not be hasty to draw firm conclusions where human destines are at stake”.

(Ceci 1996, p. 247)

## Data Availability

The original contributions presented in the study are included in the article, further inquiries can be directed to the corresponding author.
